# In Vitro and In Vivo Wound Healing Activity of *Astragalus floccosus* Boiss. (Fabaceae)

**DOI:** 10.1155/2022/7865015

**Published:** 2022-03-29

**Authors:** Fatemeh Akbari, Mohammad Azadbakht, Ali Bagheri, Lale Vahedi

**Affiliations:** ^1^Department of Pharmacognosy, Mazandaran University of Medical Sciences, Sari, Iran; ^2^Department of Plant and Animal Biology, University of Isfahan, Isfahan, Isfahan, Iran; ^3^Department of Pathology, Mazandaran University of Medical Sciences, Sari, Iran

## Abstract

Estrogens are a group of sex hormones which have receptors on the skin and lead to increased cells and wound healing. Normally isoflavonoids are present in *Astragalus floccosus* Boiss. (Leguminosae). Therefore, the present study was conducted to evaluate the presence of isoflavonoids in *A. floccosus'* rich fraction of flavonoid and evaluate its wound healing effect accordingly. Flavonoids were evaluated by LCMS. Scratch was conducted and the medium culture was treated with the *Astragalus'* rich fraction of flavonoid (RFF) and was compared with nontreated culture during 48 hours. In addition, in vivo full-thickness wound healing evaluation was performed on rats. The rats were put into four groups and treated on a daily basis for 21 days with a cream containing 1.5% of the RFF (group 1), silver sulfadiazine (group 2), and Vaseline (group 3) separately. The nontreated group (group 4) was created for a better comparison. During the examination, wound size was evaluated and histopathological examination was performed. Herbal analysis detected 11 flavonoids, including 2 isoflavonoids, Calycosin-7-O-beta-D-glucoside and Formononetin, in the RFF. In vitro scratch wound healing showed significant improvement with RFF treatment in comparison to nontreated medium. Furthermore, in vitro drug release of *Astragalus* ointment showed a stationary line during 24 h and 0.14 mg/ml of flavonoid penetrated the skin. In vivo wound size evaluation showed significant improvement in the group treated with the RFF in comparison to other groups. Histopathological results indicated that congestion, edema, inflammation, necrosis, and angiogenesis decreased during the examination and fibroblast proliferation fibrosis epithelization was increased especially in the RFF group in comparison to the silver sulfadiazine and free groups. In conclusion, *A. floccosus* showed that wound healing activity in both in vitro and in vivo analyses can be attributed to the presence of isoflavonoids with estrogen-like activity in this plant.

## 1. Introduction

Wound is a complication which affects people and has high prevalence. It consists of four biological processes: hemostasis, inflammation, proliferation, and remodeling [1]. Both dermal and immunity cells are involved in the process. Examples of immunity cells are neutrophils, macrophages, and lymphocytes, and dermal cells are keratinocytes, fibroblasts, and endothelial cells [2].

Local and systematic factors affect wound healing, which are inseparable. Local factors are venous sufficiency, foreign body, infection, and oxygenation [3]. Systematic factors are underlying diseases, medications, aging, sex hormones, and any condition that suppresses the immune response. Sex hormones including estrogens (estrone and 17*β*-estradiol) and androgens and related hormones (testosterone, 5*α*-dihydrotestosterone, and dehydroepiandrosterone) affect the wound healing process [4]. The difference in gene expression between the wounds of aged and young individuals is regulated almost exclusively by estrogen [5]. Androgens, however, have a negative effect on wound healing [6].

Estrogens play a role in wound healing by regulating a variety of genes involved in regeneration, matrix production, protease inhibition, epidermal function, and genes that are primarily associated with inflammation [7].

Isoflavonoids are classes of flavonoid which have the basic 3-phenylchromen-4-one structure; they are found in two forms: glycoside and nonglycoside (aglycone). Normally, they are found in glycoside form. These compounds have estrogen-like structures. Isoflavonoids can bind to the human estrogen receptor (ER) and exhibit partial agonist effects. These compounds have antiatherogenic and cardiovascular protective effects, increase the synthesis of vitamin D in nonrenal cells, stimulate calcium uptake in bone, and increase the production and differentiation of bone cells. These effects can lead to improved bone health, especially in the postmenopausal period. Isoflavonoids may alter endogenous estrogens metabolism and may have antiproliferative and anti-breast-cancer effects. Studies have shown that a diet high in isoflavonoids can delay the development of prostate cancer and can improve postmenopausal symptoms such as flushing and headaches [8, 9].

Isoflavonoids are known to be present in Leguminosae (Fabaceae) family. One of the largest genera in this family is *Astragalus* with more than 3000 species in the world, accounting for more than 10% of the flora of Iran [10]. The root of this genus has traditionally been used to improve resistance to, and help with, immunological disorders and viral infections, liver protection, antiperspirants, diuretics, and tonics and also for treatment of a wide range of diseases such as diabetes mellitus, high blood pressure, leukemia, nephritis, and uterine cancer and to accelerate the healing speed of bums and abscesses [11].

In the current study, in vitro and in vivo effects of an endemic of native Iranian *Astragalus* species, namely, *A. floccosus* Boiss., on wound healing will be analyzed and pharmacological efficacy of this extract will be discussed based on phytochemical profile.

## 2. Materials and Methods

### 2.1. Herbal Preparation

#### 2.1.1. General Experimental Procedures and Materials

Plants were collected from the growing areas of this plant species in the western parts of Isfahan province (lattitude 32° 58′N; longitude 50° 01′E) and identified by relevant literature [12, 13]. Next, some herbarium samples were prepared and kept in the herbarium of the Department of Pharmacognosy of Mazandaran University of Medical Sciences and also the herbarium of University of Isfahan (HUI) (herbarium code: 23019).

About 3 kilograms of root of *A. floccosus* was extracted by maceration with absolute methanol at room temperature for 3 days and repeated three times and the extract was dried under vacuum by rotary evaporator followed by freeze drier. The extract was poured into a special container and stored in a −30°C freezer.

### 2.2. Heavy Metal Analysis

Extract in solution with a ratio of 1 : 3 of nitric acid and hydrochloric acid was digested. The extract was put in the oven at a temperature of 460°C for 24 h. The ash was digested in 10 ml of 1HNO3: 3HCl solution on a hot plate. Then, it was centrifuged and treated with 1% HNO3 up to the 25 ml volume. Determination of the cadmium, lead, and chromium elements was carried out with Flame Atomic Absorption Spectrometry (MP-AES 4100: microwave atomic emission spectroscopy, Agilent, Australia) compared with the standard solution of each element [14].

### 2.3. Analysis of the Extract

#### 2.3.1. Flavonoids and Total Phenolic Content Determination

The spectrophotometry method was used for evaluation of flavonoid and phenol content with the aluminum chloride colorimetric method and the Folin-Ciocalteu assay method, respectively [15].

#### 2.3.2. Tannin Determination

1 mg/ml of extract was mixed with 100 mg and kept for 15 minutes in refrigerator and then centrifuged with 3000 rpm for 10 minutes. 0.5 ml of the supernatant solution was removed, and the phenolic absorbance was measured with Folin-Ciocalteu assay method. The difference in the initial phenol absorption and secondary absorbance indicates the amount of tannin [16].

#### 2.3.3. Preparation of Flavonoid-Rich Fraction

40 g *Astragalus* extract was monitored by TLC for fractionation; due to polar constituencies, the extract was partitioned with EtOAc (18 g) and MeOH (22 g). Each fraction was analyzed by UV spectrophotometry and the aluminum chloride colorimetric method based on quercetin to find rich fraction of flavonoid (RFF).

### 2.4. Sample Preparation for LC-MS Study

The RFF was dissolved with the minimum amount of water and then filtrated with a 0.45 *μ*m pore size filter. 10 *μ*l of RFF was injected to Agilent 6100 Quadrupole LC/MS System to detect flavonoids, which were checked with library standard chromatogram in the system using the diode array detector SL (micro flow cell: 2 *μ*L, 3 mm path length) [17].

### 2.5. LC-ESI-MS Instrumentation

LC-MS was conducted according to Lin method [17]. For this purpose, an Agilent series 6100 LC/MS system (Agilent Technologies, Santa Clara, CA, USA) with photodiode array detector was set at 260 nm. The UV spectra were examined with a range of 200 nm to 500 nm to obtain the maximum absorbance wavelength. A 150 mm × 3.0 mm, 3.5 *µ*m Waters XTerra MS with a sentry guard column (Symmetry C18, 5 *μ*m, 20 × 3.9 mm) was used. The mobile phase contained (1) water with 0.25% (v/v) acetic acid and (2) acetonitrile with 0.25% (v/v) acetic acid using linear gradient of 17–42% (v/v) (2) for 38 minutes. The temperature and flow rate were set to 45°C and 0.2 ml/min, respectively.

The LC system was joined to the mass spectrometer directly without stream splitting and, by using an electrospray interface Model HP 59987A, the ESI-MS spectra were obtained from the positive ion mode. The nebulizer pressure (N2) was 5.5 × 105 Pa and temperature of dying gas (N2) was set to 350°C, with a gas flow rate of 40 ml/min [17].

### 2.6. In Vitro Drug Release Study

**Figure 1:**
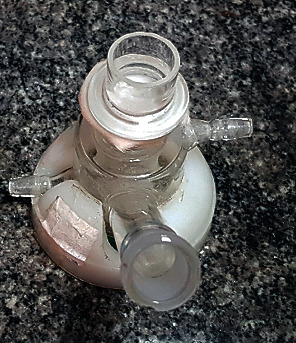
Dialysis tube.

Two rats were anesthetized and their abdominal skin hair was removed. The abdominal skin was separated and held in normal saline for 12 h and was then applied to the dialysis tube. *Astragalus* RFF ointment was applied on the surface of the dialysis tube and the lower surface was exposed to deionized water hermetically sealed and soaked in a tube containing 35 ml of deionized water (Figure 1). Continuous shaking was applied with heater stirrer and a magnet in inner part, and the temperature was set to 32°C ± 0.5°C at 2, 4, 6, 8, 10, 12, and 24 times. After starting the procedure, 5 ml sample was given from the solution and 5 ml of deionized water was placed in the dialysis tube to keep the sink situation. The sample evaluated with spectrophotometer at 415 nm based on quercetin has the maximum wavelength [18].

### 2.7. In Vitro

#### 2.7.1. MTT, Cell Viability Assay

Human dermal fibroblast (HDF) cell was purchased from Royan Institute for Stem Cell Biology and Technology and cells were seeded in a 96-well microplate (1 × 104 per well) in RPMI-1640 culture medium supplemented with a 10% fetal bovine serum (FBS), 0.01 M of HEPES (4-[2-hydroxyethyl]-1-piperazineethanesulfonic acid), 1% mixture of antibiotic/antimycotic, 0.001 M of sodium pyruvate, and 0.02 M of L-glutamine and then incubated in an incubator with a humidified atmosphere with 5% CO_2_ for 24 h.

After 24 h, the medium was changed with the above medium which was supplemented with RFF with different doses of 2000, 1000, 500, and 125 µ/ml.

MTT assay was performed after 24 and 48 h. Therefore, 10 µl of MTT reagent (3-[4,dimethylthiazole-2-yl]-2,5-diphenyl tetrazolium bromide) was added to the microplate and incubated for 4 h and 90 µl of formalin buffer was added to each well. Furthermore, 100 µl 10% SDS in 0.01 M HCl as solubilization solution was added to each well and remained inside the incubator at 37°C. After solubilization of purple formazan product completed over night, microplates absorbance value was measured at 570 nm wavelength with ELISA reader [19].

A1:

Viability percentage of cell = (absorbance of RFF treated cultures − absorbance of background control)/(absorbance of control cultures − absorbance of background control) × 100.

### 2.8. Evaluation of the In Vitro Wound Healing Activity

#### 2.8.1. Cell Culture

NHDF cell line was maintained in RPMI-1640 culture medium with supplemented material including 10% FBS, 1% mixture of antibiotic/antimycotic, 0.01 M of HEPES, 0.02 M of L-glutamine, and 0.001 M of sodium pyruvate. Finally, it was incubated in an incubator (at 37°C and a humidified atmosphere with 5% CO_2_) [20].

#### 2.8.2. Wound Scratch Assay

In vitro wound healing activity of the RFF was evaluated by wound scratch assay [21]. Cells of normal human dermal fibroblast (NHDF) were seeded in 6 wells of 12-well plates (4 × 104 cells/well) and cultured with the above condition to obtain monolayer cells in each plate. Cells adhered to the plate and then the medium was eliminated from the well and central scraping was performed using the tip of the p200 micropipette. The wells were washed with PBS to remove the nonadherent cells and the cell residue after scratching.

The specific dose of *Astragalus* RFF was selected after MTT assay to treat the wells. Thereby, the selected dose of *Astragalus* was mixed and dissolved in RPMI-1640 and added to the wells. For the control group, three wells were treated with supplemented RPMI-1640 culture medium. The plates were incubated at 37°C (5% CO_2_) and, in the period of 0, 2, 24, and 48 hours, the scratches were evaluated under the microscope and the progress of wound healing of RFF was evaluated and compared to the control group and was calculated manually by IC Measure software version 2.0.0.286 (The Imaging Source, Germany) [21].

### 2.9. In Vivo

#### 2.9.1. Preparation of the Herbal Ointment

For preparation, 1.5% ointment of *Astragalus* RFF and 7.5 g of RFF were dissolved in the least possible amount of water and added to the minimum amount of Eucerin up to homogenate and all were gradually added to the ointment base Vaseline up to 500 g.

#### 2.9.2. Animals

Healthy male rats weighing between 200 and 220 g were taken from the Institute for Laboratory Animal Research of Mazandaran University of Medical Science. Animals have free access to food and water and are kept under standard conditions (12-hour light-dark cycle and room temperature). Animal rights were respected according to the principles of the Association for the Protection of Animal Rights [18].

This study was conducted in accordance with the principles of the Association for the Protection of Animal Rights with the ethical IR.MAZUMS.REC.1400.8171 and animal rights were respected.

### 2.10. Experimental Wounding

Animals were anesthetized with ketamine-xylazine mixture with a dose of 0.1 ml/100g per body weight via intraperitoneal injection. The dorsal hairs of animals were cut into 2.5 × 2.5 cm circular shapes; then, full thickness wound was made with an excision on the back of all animals [22].

#### 2.10.1. Animals and Experimental Design

40 rats were divided into four groups (*N* = 10, each animal kept in separate cage); group one was topically treated with ointment base Vaseline; group two was topically treated with a 1.5% RFF of *Astragalus* ointment; group three was topically treated with silver sulfadiazine as the standard medicine; and group four did not receive anything as the normal control.

### 2.11. Wound Analysis

Wound dimensions were measured on days 1, 4, 7, 11, 14, and 21. For this purpose, the wound sizes were drawn on transparent paper and transferred to graph paper. The ratio of wound healing was calculated according to the following formula [18]:

A2:

The ratio of wound healing = (area of original wound − area of remaining wound)/area of original wound × 100.

#### 2.11.1. Wound Microbial Assay

One rat from each group was randomly selected and the rats' wounds were sampled with sterile swabs on days 6 and 20 to measure the microbial load of the wounds. The specific media cultures for *Staphylococcus aureus* (soybean casein digest agar), *Pseudomonas aeruginosa* (nutrient agar), and *Candida albicans* (Sabouraud dextrose agar) were prepared. The mediums after sampling were incubated at 34-35°C for bacterial and at 24°C for fungal growth evaluation [23].

### 2.12. Tissue Collection

On days 7 and 14, six rats of each group were euthanized by chloroform inhalation and the tissues were collected. Also, normal healthy skin of rats was removed for further comparison. All samples were kept in separate containers filled with 10% formalin and stained with hematoxylin and eosin (H&E) and Masson's trichrome stain (MT). Samples were examined for congestion, edema, necrosis, fibroblast proliferation, collagen formation, angiogenesis, and epithelialization.

### 2.13. Evaluation of Biochemical Parameters

#### 2.13.1. DNA Estimation

On days 4 and 19, one rat from each group was euthanized and wound tissues were removed and kept in separate containers and hydrated with normal saline and kept in freezer with temperature of −30°C. Total DNA quantities were evaluated using the phenol chloroform method and measured with the microvolume spectrophotometer, NanoDrop 2000/c (Thermo Fisher Scientific, USA) [24].

### 2.14. Statistical Analysis

Statistical analyses were performed using SPSS 26 software. A significance level of 0.05 (*P* ≤ 0.05) was considered in all cases, and all results were represented by Mean ± SEM. The variable's normality was assessed using the Shapiro-Wilk test. One-way ANOVA test and Tukey's post hoc test were used. The nonparametric Kruskal-Wallis test analysis was performed when the data were not normally distributed.

## 3. Results

### 3.1. Heavy Metal

Heavy metal results showed presence of lead and chromium with amounts of 3.5 and 5.01 mg/kg, respectively, and the amount of cadmium was lower than 0.01 mg/kg.

### 3.2. Phenol, Flavonoid, and Tannin of the Extract

By standard curve equation of quercetin (*y* = 0.0066*x*+0.2367, *R*^2^ = 0.9712), the amount of flavonoid of extract was calculated and, by taking the extraction efficiency (9%) into account, it was calculated as 0.67 g per 1 g of the root of *A. floccosus.* The total amount of phenol was calculated based on gallic acid standard curve equation (*y* = 0.0199*x*+0.0752, *R*^2^ = 0.9914) and it was 0.67 mg per 1 g *A. floccosus* root. According to the difference in the amount of phenol before and after the addition of PVP, the amount of precipitated tannin was calculated and it was 0.031 g per 1 g of the root of *A. floccosus.*

### 3.3. *Astragalus* Rich Fraction of Flavonoid

The total flavonoid in the EtOAc and MeOH was measured and EtOAc was selected due to higher amount of flavonoids (0.446 g per 1 g of the root of *A. floccosus.*)

### 3.4. LC-MS Evaluation

**Figure 2:**
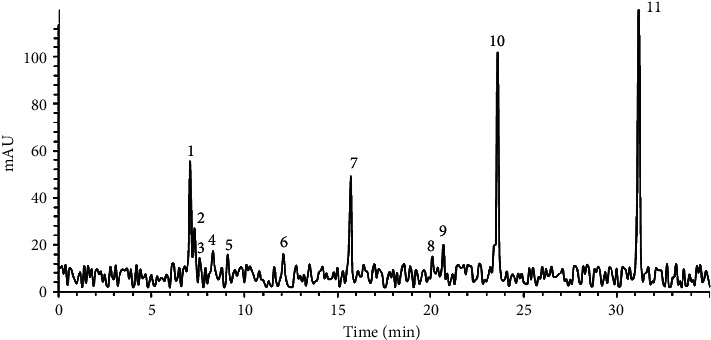
LC-MS analysis of the root of *A. floccosus*.

The *Astragalus* RFF LC-ESI-MS analyses are shown in Figure 2. The identification of individuals peak, retention time, concentration, and [*M*+*H*]+ are listed in Table 1. Based on a comparison of those values with system library data, 11 flavonoid peaks were identified which are shown in Figure 2 and Table 1. Maximum concertation belonged to Formononetin and Kaempferol-3-O-glucoside which were isoflavone and flavanol, respectively.

### 3.5. In Vitro Drug Release Study

**Figure 3:**
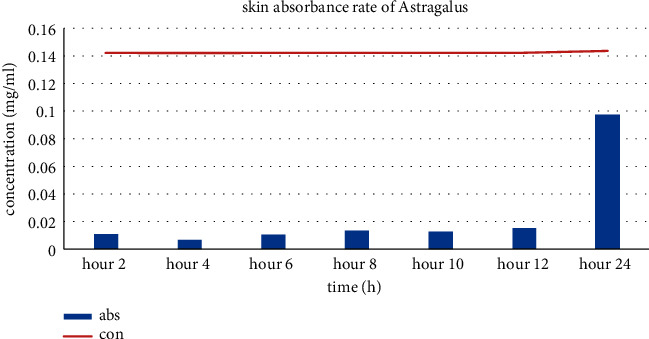
Skin absorbance ratio. The orange line shows the concentration of RFF formula penetrating the membrane during 24 h.

The quercetin absorbance at 415 nm was *y* = 0.0166*x* + 0.142, *R*^2^ = 0.996. Using the same formula, the flavonoid penetrated the membrane at a constant rate and the amount was about 0.14 mg/ml during the examination (Figure 3).

### 3.6. MTT Assay

**Figure 4:**
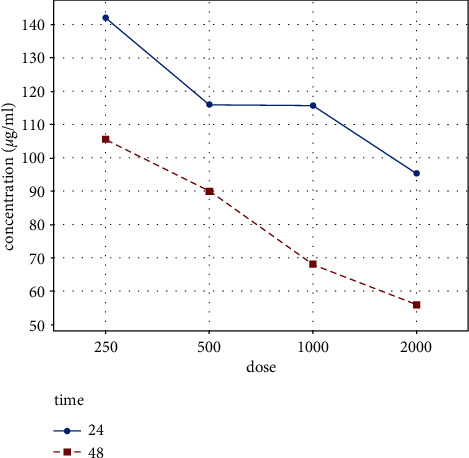
Percent of NHDF cell viability treated with different doses of 2000, 100, 500, and 250 µg of RFF.

Cell viability results indicated that the RFF with a dose of 2000 µg was toxic after 48 h (0.97% cell viability), and, with doses of 250, 500, and 1000 µg, cell viability was over 100 percent which indicated cell growth. Maximum cell viability percentages were reached at doses of 250 µg and 500 µg, respectively, in both 24 and 48 h (Figure 4). With these results, the dose of 500 µg was selected for scratch wound healing study.

### 3.7. Evaluation of In Vitro Wound Healing Activity

**Figure 5:**
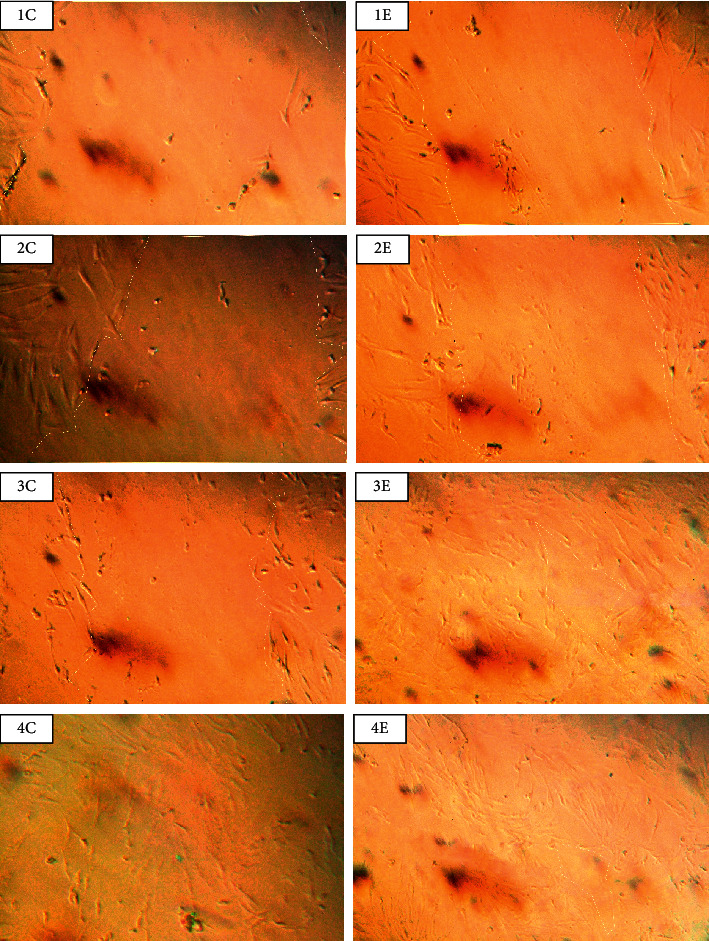
microscopic images (100×) of scratch wound healing assay. Pictures 1, 2, 3, and 4, respectively, represent 0, 2, 24, and 48 hours after treatment. (E) is RFF and (C) is control sample.

**Figure 6:**
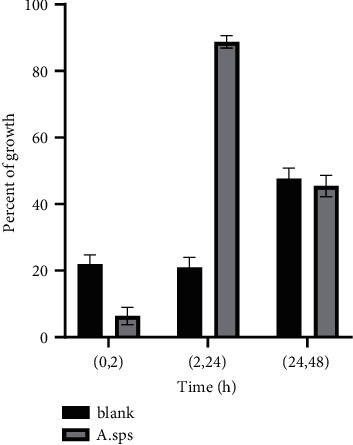
Percent of growth during the experimental procedure. Between 2 and 24 h, the RIFF group has the maximum effect (89.45% of scratch closed).

In general, the wound healing results obtained from RFF samples showed no mortality. Furthermore, an increase in normal fibroblast cells was observed. By comparing the microscopic images of the RFF and control samples, it could be seen that the RFF group shows better efficacy at 24 h after treatment. No other significant differences were observed (Figures 5 and 6 and Table 2).

### 3.8. Wound Microbial Assay

The observations show that although the *Staphylococcus aureus* was not seen in the free, RFF, and Vaseline medium on day 6, all groups showed microbial colonization on day 20. NA mediums showed the presence of *Pseudomonas aeruginosa* on day 6 but after day 20 all mediums were infected with colonies of this microorganism. Wound sample analysis for *Candida albicans* showed the presence of *Candida* in silver and RFF groups on day 6 but it is not seen in the RFF medium after day 20 and in the silver medium, as the number of colonies reduced. In addition, the Vaseline group had been infected on day 20 (Table 3).

### 3.9. Wound Analysis

**Figure 7:**
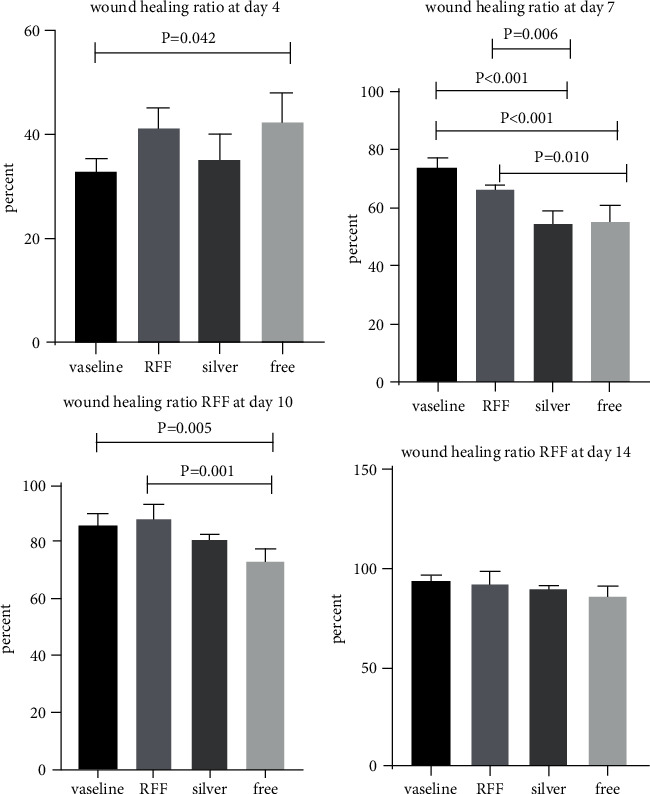
Wound healing ratio in silver, free, Vaseline, and RFF groups during the study on days 4, 7, 10, and 14.

Wound evaluation showed that, by the fourteenth day, 90% of the wound has healed in all groups. The wound healing ratio in free groups in the beginning was the highest but, over time, all groups sped up in wound healing and showed better effects compared to the free group. RFF group had the second highest wound healing activity on day 4 and the highest activity on day 7 but the Vaseline group showed better effects on days 10 and 14 in comparison to the other groups. To follow the pairwise comparisons of the groups, this study used Tukey's test and the results showed that the wound healing ratio on the 7th day of the RFF was higher than those in the silver (*P* = 0.006) and free group (*P* = 0.010). Furthermore, after the study group was treated, the RFF showed minimum wound scar (Figure 7 and Table 4).

### 3.10. Wound DNA Estimation

**Figure 8:**
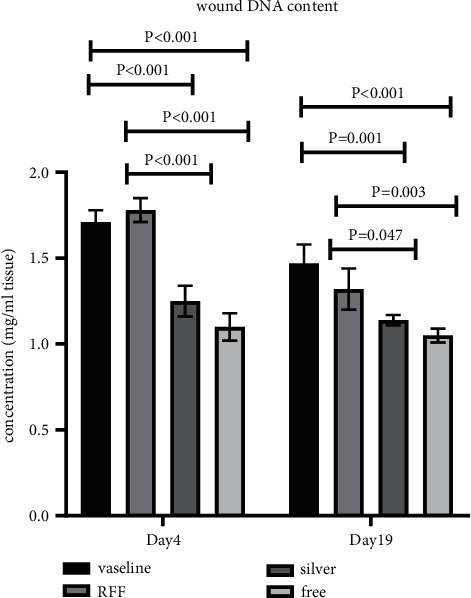
Total DNA evaluation between groups on days 4 and 19.

Total DNA evaluation showed that, with the same weight of wound tissue, the groups on day 4 had a higher DNA compared to day 19. RFF group on day 4 had shown the highest amount of DNA between groups and, on day 19, all groups almost reached the stationary phase with a constant amount of DNA content. On day 4, the free group had the minimum level of total DNA, and day 19 showed the maximum DNA content in comparison to other groups (Figure 8).

### 3.11. Histopathological Examination

Histopathological results indicated that congestion, edema, inflammation, necrosis, and angiogenesis decreased during the examination and fibroblast proliferation; fibrosis and epithelization were increased especially in the RFF group in comparison to silver sulfadiazine and free group. According to the Kruskal-Wallis test, epithelialization, the proliferation of fibroblast, fibrosis, angiogenesis, congestion, edema, inflammation, and necrosis were significant among groups (*P* = 0.002). On day 4, fibroblast proliferation in the RFF group was more than that in silver group (*P* = 0.006), that in the free group was more than that in the silver group (*P* = 0.006), that in the RFF group was more than that in the Vaseline group (*P* = 0.038), and that in the free group was more than that in the Vaseline group (*P* = 0.038); fibrosis in alpha group was more than those in silver group (*P* = 0.033) and Vaseline group (*P* = 0.033). On day 14, the postoperative test showed that epithelialization was higher in the RFF group than in Vaseline group.

In Masson's trichrome dye samples, blue zone showed proliferation of tissue which can be attributed to collagen bond formation. On day 14, the Vaseline and RFF groups showed higher blue zone (Table 5).

## 4. Discussion

Wound is a complication which affects people and the treatment becomes more important when it is associated with many factors such as length and depth of the wound, wound infections, and underlying diseases like diabetes [24].

Infection is one of the important local factors. Microorganisms normally exist in the surface of skin. When the skin is wounded, they have access to underlying tissue. Factors such as state of infection, replication status, and microorganism loading in the tissue represent the classification of wound infection such as microbial contamination, colonization, local infection, and systematic separation invasive infection [25].

Inflammation is body's normal response to the wound healing process, and microbial contamination prolongs this response. Both bacteria and endotoxins elevate proinflammatory cytokines such as TNF-*α* and interleukin-1. Furthermore, inflammation increases protease enzyme in the tissue which degrades growth factors rapidly [26, 27]. If this situation continues in the wound, it leads to failure in healing*. Staphylococcus aureus (S. aureus)* and *Pseudomonas aeruginosa (P. aeruginosa)* are important causes of wound infection [28]. In the current study, *Staphylococcus aureus* and *Pseudomonas aeruginosa* were not observed on day 6 in any group except standard medicine, and on day 20 all groups showed microbial colonies. Therefore, although there was colonization of bacteria, wound healing was better completed in the RFF group than in all the other groups. It means that the RFF modulated the body inflammation responses.

Moreover, DNA evaluation of wound indicated that, on day 4, total DNA was higher than that on day 19 according to higher accumulation immunity and inflammation responses and with a faster rate in RFF group in comparison with other groups but on day 19 all groups reached the constant level of total DNA which meant the healing process was about to finish or was already over.

It can be concluded that when wound healing was near completion, the new tissue was replaced instead of inflammation response; this finding is similar to Tottoli et al.'s study [29]. On day 4, total DNA of the RFF is higher in comparison to other groups which indicated that the RFF group evoked immunity response and accumulation of wound repairing cells for treatment. But it can be concluded that, in the free group, this response was delayed and, on day 19, DNA content was increasing.

Moreover, the RFF treated group showed antifungal activity according to *Candida albicans* observed in the RFF group medium on day 6 which was not seen on day 19.

*Astragalus* species belong to Legumes family and are reported to have polysaccharides, saponins, and isoflavonoids [30, 31]. Many species were used because of their immunomodulatory activity for inflammatory disorders. Studies indicated that immunomodulating activity in this family is related to isoflavonoids and specially polysaccharide. In the current study, herbal analysis of the *A. floccosus* showed high amounts of flavonoid and phenolic components content and LC-MS evaluation represented some flavonoid and glycoside isoflavonoids with base structure of rutin, quercetin, Kaempferol, and Calycosin.

Furthermore, isoflavonoids are a group of compounds which have estrogen-like activity and give the plant estrogen-related properties. Studies reported that elderly individuals' rate of wound healing decreased. Moreover, the rate of wound healing was higher in females in comparison to males. Further studies reported that this observation is directly related to estrogen hormones [32]. Further research have shown that estrogens increased mitotic activity in the *epidermis* [33].

Other studies evaluated the effect of *Astragalus membranaceus* (Fisch.) Bunge wound healing effect in vitro and in vivo and, in both conditions, it showed wound healing activity [34, 35]. Rats, mice, and guinea pigs were usually used for wound healing assay in the in vivo condition.

The rate of drug delivery of the ointment was measured by the amount of flavonoid crossing the skin. In this study, Vaseline based ointment crossed the skin with constant amount or, in other words, zero kinetic absorbance. Due to the fact that Vaseline cannot be removed easily from dorsal skin of rats, the duration of contact of the RFF with the wound is longer and the healing process is completed better.

In the present study, topical application of *Astragalus* significantly enhanced the rate of wound healing as assessed by the increase in collagen synthesis wound tissues, a finding which was similar to Qian et al.'s study. Histological findings also showed enhanced proliferation of fibroblasts and epithelialization and they were significantly better in RFF group than in silver and free groups. Shukla et al. measured the hydroxyproline levels of wound tissues such as biomarker of amount of collagen (cellular repair building materials) [36]. In this current study, collagen was evaluated by Masson's trichrome stain and fibrosis indicated collagen bonds.

Afonso et al. evaluated the in vitro wound healing activity by scratching assay on NHDF cell lines [37]. The rate of closing the wound was measured and cells migration demonstrated wound healing. In the current study, the dose of treatment of medium was selected based on toxicity evaluation by MTT assay. Obviously, wound migration and closing the scratch were observed especially in RFF group 24 hours after treatment with *Astragalus* RFF in comparison with control samples which indicated a faster rate of wound healing.

Overall, proinflammatory analysis of cytokines for direct evaluation of wound inflammation is suggested for the next studies. It is suggested to evaluate isoflavonoids, saponins, and polysaccharide in *Astragalus floccosus* Boiss. to analyze the specific efficacy of each one of them on wound healing.

## 5. Conclusion

*Astragalus floccosus* Boiss. rich fraction of flavonoid has a high affinity to bind to estrogen receptors alpha and beta which are present in the skin tissue and play role in wound therapy. This study showed that RFF has a wound healing activity in in vitro and in vivo conditions with low toxicity. This extract can be considered as an adjuvant therapy for skin wounds due to the presence of compounds (especially isoflavonoids) and can be used for further clinical and commercial evaluations. Due to the limitation of our study to isolate isoflavone glycoside from the extract, it is better to evaluate each one of the LC/MS result compounds for wound healing analysis in future studies.

## Figures and Tables

**Table 1:** The LC-MS peaks information, retention time (RT) values, [*M*+*H*]^+^, and concentration.

Peak IDs	RT (min)	Recovery (%)	m/z [*M*+*H*]^+^	Concentration (µg/g)
1. Calycosin-7-O-beta-D-glucoside	7.08	101	447	83.26278
2. 7,4′-Dihydroxy-3′-methoxyflavone 7-glucoside	7.31	97	446	40.55473
3. 3′-O-Methylorobol-7-O-glucoside	7.58	98	447	21.7406
4. Quercetin-3-O-sophoroside	8.31	97	636	26.05643
5. Quercetin 3-O-neohesperidoside	9.1	98	609	23.80803
6. Kaempferol-3-O-neohesperidoside	12.1	99	770	24.32915
7. Kaempferol-3-O-neohesperidoside	15.72	99	594	73.79588
8. Kaempferol-3-O- neohesperidoside-7-O-rhamnoside	20.1	93	595	22.56475
9. Kaempferol-3-rhamnoside-(1->2)-rhamnoside	20.7	99	593	29.87147
10. Kaempferol-3-O-glucoside	23.6	98	593	152.6532
11. Formononetin	31.19	95	269	198.0895

**Table 2:** Calculation of scratch area and percentage of growth at 0, 2, 24, and 48 hours after treatment. Percent of growth = (T2 scratch area − T1 scratch area)/T1 scratch area × 100.

Time (h)	Area		Percent of growth	
	RFF	Blank	RFF	Blank
0	627.5	888.23	9.73%	25.02%
2	566.4	666.05		
			89.45%	17.24%
24	59.75	551.18		
			49.94%	54.20%
48	29.9	252.41		

**Table 3:** Groups wound microbial analysis on days 6 and 20.

Groups	Day	SCDA (*Staphylococcus aureus*)	NA (*Pseudomonas aeruginosa*)	SDA (*Candida albicans*)
Silver	6	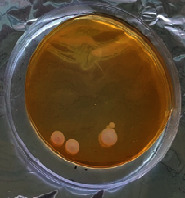	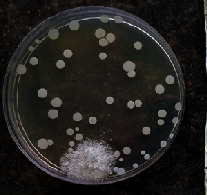	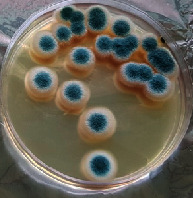
	20	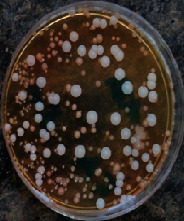	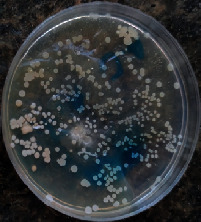	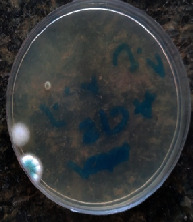
Free	6	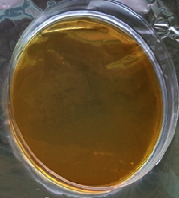	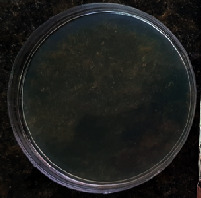	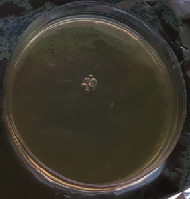
	20	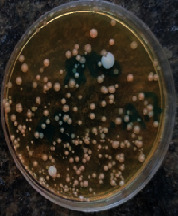	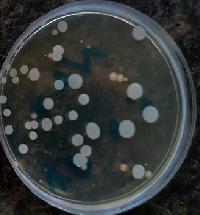	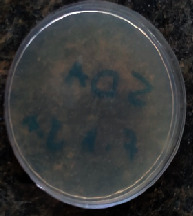
RFF	6	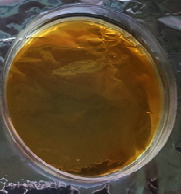	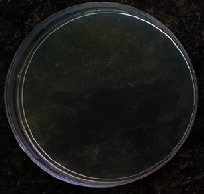	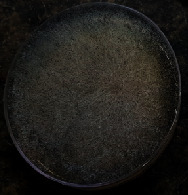
	20	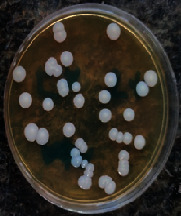	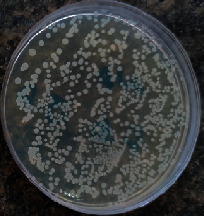	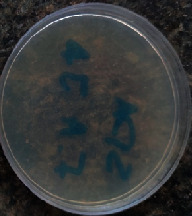
Vaseline	6	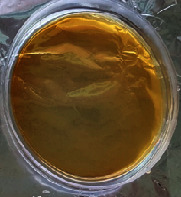	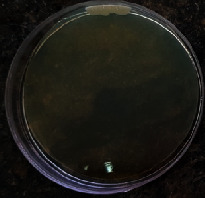	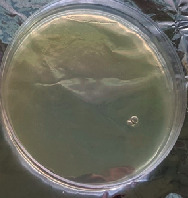
	20	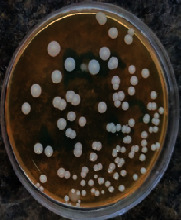	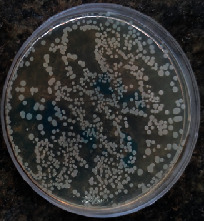	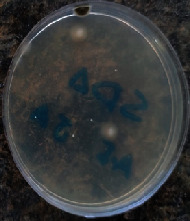

**Table 4:** Wound analysis in RFF, Vaseline, silver, and free groups on days 1, 4, 7, 11, 14, and 21 (the full thickness wound induced in all groups at day one and there is no difference between groups).

Day	RFF	Vaseline	Silver	Free
1	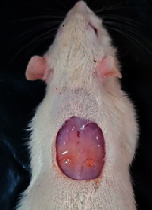	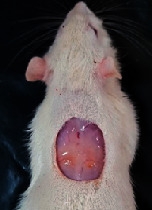	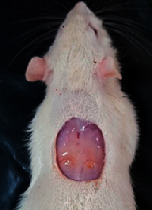	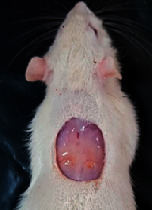
4	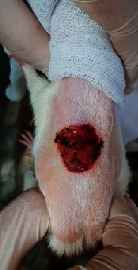	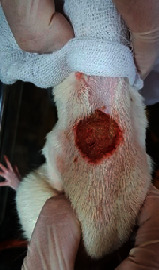	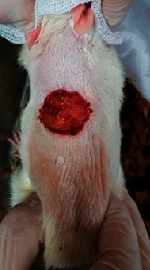	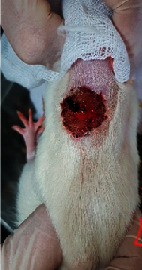
7	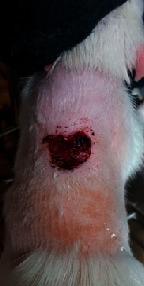	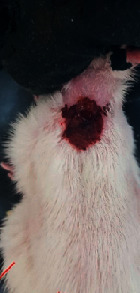	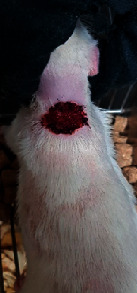	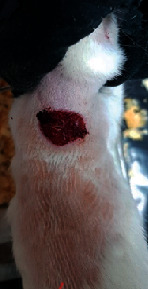
11	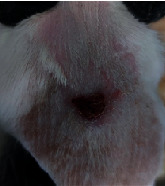	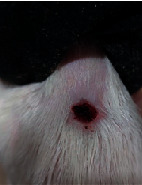	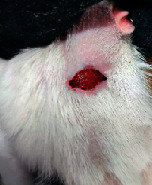	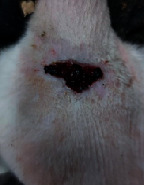
14	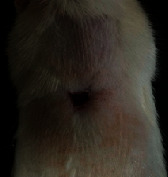	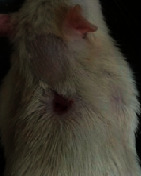	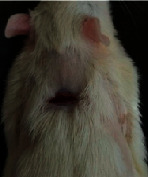	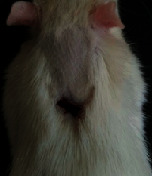
21	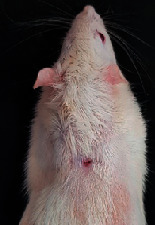	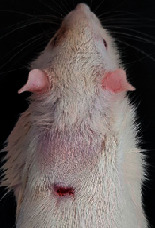	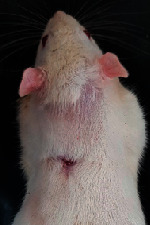	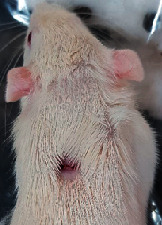

**Table 5:** Microscopic images (40×) of different treatment groups tissue samples after being stained with H&E and MT on days 7 and 14 after treatment (blue zone indicates the fibroblast proliferation). Squares represent morphology changes such as edema, congestion, inflammation, necrosis, and angiogenesis during experimental period.

Groups	Day 7		Day 14	
	H&E	MT	H&E	MT
Vaseline	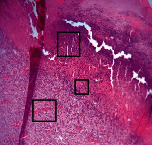	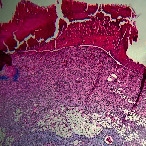	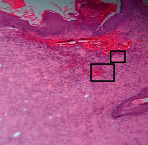	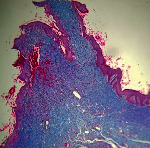
RFF	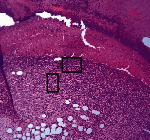	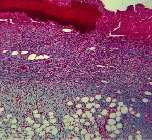	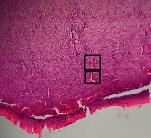	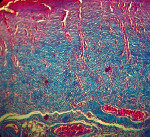
Silver	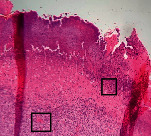	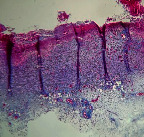	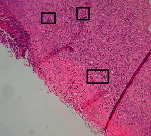	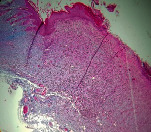
Free	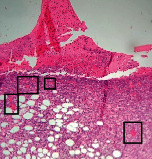	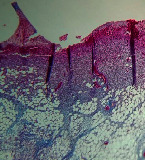	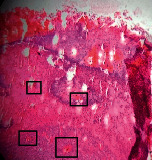	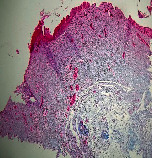

## Data Availability

All materials such as rats, silver sulfadiazine, Vaseline, microbial medium cultures, and ethanol for extraction were provided by Mazandaran University of Medical Science and data were obtained from the laboratories of Mazandaran University of Medical Science such as pathology and serology laboratories.
